# Perm1 regulates cardiac energetics as a downstream target of the histone methyltransferase Smyd1

**DOI:** 10.1371/journal.pone.0234913

**Published:** 2020-06-23

**Authors:** Shin-ichi Oka, Amira D. Sabry, Amanda K. Horiuchi, Keiko M. Cawley, Sean A. O’Very, Maria A. Zaitsev, Thirupura S. Shankar, Jaemin Byun, Risa Mukai, Xiaoyong Xu, Natalia S. Torres, Anil Kumar, Masayuki Yazawa, Jing Ling, Iosif Taleb, Yukio Saijoh, Stavros G. Drakos, Junichi Sadoshima, Junco S. Warren

**Affiliations:** 1 Department of Cell Biology and Molecular Medicine, Rutgers New Jersey Medical School, Newark, NJ, United States of America; 2 Nora Eccles Harrison Cardiovascular Research and Training Institute, University of Utah, Salt Lake City, UT, United States of America; 3 Department of Cardiology, Ningbo Medical Center Lihuili Hospital, Ningbo, Zhejiang, China; 4 Metabolic Phenotyping Core Facility, University of Utah, Salt Lake City, UT, United States of America; 5 Columbia Stem Cell Initiative, Rehabilitation and Regenerative Medicine, Columbia University, New York, NY, United States of America; 6 Pharmacology, Columbia University, New York, NY, United States of America; 7 Division of Cardiovascular Medicine, Department of Internal Medicine, University of Utah School of Medicine, Salt Lake City, UT, United States of America; 8 Department of Neurobiology and Anatomy, University of Utah, Salt Lake City, UT, United States of America; 9 Institute of Resource Developmental and Analysis, Kumamoto University, Kumamoto, Japan; Niigata Daigaku, JAPAN

## Abstract

The transcriptional regulatory machinery in mitochondrial bioenergetics is complex and is still not completely understood. We previously demonstrated that the histone methyltransferase Smyd1 regulates mitochondrial energetics. Here, we identified *Perm1* (PPARGC-1 and ESRR-induced regulator, muscle specific 1) as a downstream target of Smyd1 through RNA-seq. Chromatin immunoprecipitation assay showed that Smyd1 directly interacts with the promoter of Perm1 in the mouse heart, and this interaction was significantly reduced in mouse hearts failing due to pressure overload for 4 weeks, where Perm1 was downregulated (24.4 ± 5.9% of sham, p<0.05). Similarly, the Perm1 protein level was significantly decreased in patients with advanced heart failure (55.2 ± 13.1% of donors, p<0.05). Phenylephrine (PE)-induced hypertrophic stress in cardiomyocytes also led to downregulation of Perm1 (55.7 ± 5.7% of control, p<0.05), and adenovirus-mediated overexpression of Perm1 rescued PE-induced downregulation of estrogen-related receptor alpha (ERRα), a key transcriptional regulator of mitochondrial energetics, and its target gene, *Ndufv1* (Complex I). Pathway enrichment analysis of cardiomyocytes in which *Perm1* was knocked-down by siRNA (siPerm1), revealed that the most downregulated pathway was metabolism. Cell stress tests using the Seahorse XF analyzer showed that basal respiration and ATP production were significantly reduced in siPerm1 cardiomyocytes (40.7% and 23.6% of scrambled-siRNA, respectively, both p<0.05). Luciferase reporter gene assay further revealed that Perm1 dose-dependently increased the promoter activity of the *ERR*α gene and known target of ERRα, Ndufv1 (Complex I). Overall, our study demonstrates that Perm1 is an essential regulator of cardiac energetics through ERRα, as part of the Smyd1 regulatory network.

## Introduction

The heart requires a constant supply of energy to maintain cardiac function. Mitochondria play a central role in providing energy through oxidative phosphorylation (OXPHOS). The mitochondrial system is highly regulated by several transcription factors and coactivators that orchestrate the expression of genes involved in mitochondrial biogenesis, maintenance, and respiration capacity. Over the past few decades, our understanding of the transcriptional control of genes involved in mitochondrial bioenergetics has significantly advanced (reviewed in [1]). However, the transcriptional regulatory machinery in mitochondrial bioenergetics is complex, and it is still not completely understood how mitochondria coordinately respond to physiological and pathological stimuli.

Our recent study identified Smyd1, a muscle-specific histone methyltransferase, as a novel regulator of mitochondrial energetics in the heart [2]. We found evidence that Smyd1 activates transcription of peroxisome proliferator-activated receptor gamma coactivator 1-alpha (PGC-1α (gene name, *Ppargc1a*)), a key regulator of mitochondrial bioenergetics [2]. PGC-1α is a co-factor of several transcription factors that orchestrate the expression of genes involved in energetics, such as peroxisome proliferator-activated receptors alpha and gamma (PPARα, PPARγ), estrogen-related receptor alpha and gamma (ERRα, ERRγ), and nuclear respiratory factor 1 (NRF1) (reviewed in [1]). Cardiac-specific *PGC-1α* knockout mice develop cardiac dysfunction under basal conditions, concurrent with global downregulation of genes involved in OXPHOS [3, 4]. Thus, it has been postulated that PGC-1α is a therapeutic target to normalize mitochondrial function in heart failure. However, in a number of studies, mitochondrial impairment with downregulation of PGC-1α target genes was observed in failing hearts in which PGC-1α expression was preserved [4–8]. Moreover, maintaining PGC-1α expression during pressure overload did not show any protective effects on contractile function under these conditions [5, 9]. These studies suggest that additional transcriptional mechanisms and regulators may exist in the transcriptional regulatory machinery of mitochondrial bioenergetics.

A recent study by the Kralli group identified *Perm1* (“PGC-1 and ERR-induced regulator in muscle 1”) through screening of genes that were induced by PGC-1α and ERRs in differentiated C2C12 myoblasts [10]. Loss- and gain-of-function studies in this cell line showed that Perm1 regulates a subset of PGC-1/ERR-target genes involved in mitochondrial bioenergetics [10]. Perm1 is a striated-muscle specific protein that is predominantly expressed in the skeletal and cardiac muscle [10]. However, the role of Perm1 in cardiomyocytes has never been investigated. We show here that Perm1 is a downstream target of Smyd1 that positively regulates metabolism. We further demonstrate that Perm1 induces *ERR*α and its target genes through an ERR response element (ERRE), which is the DNA sequence critical for transcriptional control by ERRs, presumably amplifying Smyd1 regulatory signaling into metabolic networks through ERRα.

## Materials and methods

### Ethical approval

The study conformed to the National Institute of Health *Guide for the Care and Use of Laboratory Animals* (8th Edition, 2011) and was approved by the Institutional Animal Care and Use Committee of the University of Utah. Human tissue study was approved by the University of Utah's institutional review board (IRB_00030622: “Effects of Mechanical Unloading on Myocardial Function and Structure in Humans”) and informed consent was obtained from all heart failure patients and non-failing donors.

### Human myocardial tissue acquisition

Myocardial tissue was prospectively collected from the left ventricle (LV) apex at the time of cardiac transplantation, as described in our previous study [11, 12]. Control myocardial tissue samples were acquired from non-failing donors hearts that were not transplanted due to noncardiac reasons. This non-failing donor LV apical tissue was harvested and processed the same way as the failing hearts.

### Surgery

Constriction of the transverse thoracic aorta (TAC) was performed on 3-month-old wild-type (WT) male mice as previously described [4]. Mice were sacrificed 4 weeks after TAC surgery to collect cardiac tissue. WT mice that underwent a sham operation without consctrction were used as controls.

### Echocardiography

Mice at the age of 3–4 months (see S1 Table) were anesthetized using 12 μl/g of body weight of 1.75–2.5% isoflurane in 100% oxygen, and echocardiography was performed using ultrasonography (Acuson Sequoia C256, Siemens Medical Solutions USA Inc., Malvern, PA). A 13-MHz linear ultrasound transducer was used. Two-dimension guided M-mode measurements of LV internal diameter were taken from at least three beats and averaged. LV end-diastolic diameter (LVEDD) was measured at the time of the apparent maximal LV diastolic dimension, while LV end-systolic diameter (LVESD) was measured at the time of the most anterior systolic excursion of the posterior wall. LV ejection fraction and fractional shortening were calculated as follows: Ejection fraction = [(LVEDD)^3^-(LVESD)^3^]/(LVEDD)^3^ x 100; Fractional shortening = (LVEDD-LVESD)/LVEDD x 100.

### Primary cultures of neonatal rat ventricular myocytes

Neonatal rat ventricular myocytes (NRVMs) were isolated from Sprague-Dawley neonatal rats (0–1 day old) as described in our previous publication [2]. Briefly, NRVMs were obtained by enzymatic dissociation from 0–1 day old litters and plated in DMEM medium (Invitrogen, #11965) containing 1% penicillin, 1% streptomycin, 1% insulin-transferrin-sodium selenite supplement and 10% fetal bovine serum for the first 24 hours after which the cells were cultured in serum-free medium.

### RNA-seq

Total RNA was isolated from NRVMs that were treated with either siSmyd1 (Qiagen, #SI01609615, #SI01609622) or siPerm1 (Qiagen, #S102021775, #SI02021782) by a modified Trizol protocol, using the Direct-zol RNA kit miniprep plus (Zymogen, #R2071). Scrambled siRNA (scr-siRNA, Qiagen, #1022076) was used as a control. Samples were eluted in RNase-/DNase-free water. Sample quality control, library preparation, sequencing and alignments were performed by the Huntsman Cancer Institute High Throughput Genomics and Bioinformatic Analysis Shared Resource. Paired-read, 150 base pair sequencing was performed using an Illumina NovaSeq6000. The trimmed reads were aligned to the reference database using STAR in two pass mode to output a BAM file sorted by coordinates. Mapped reads were assigned to annotated genes using featureCounts version 1.6.3 [13]. The significance of changes in genes was determined by a Student’s t test of control vs. siPerm1 or control vs. siSmyd1. Genes identified as having an adjusted P value less than 0.05 in DESeq2 were considered significant. Enrichment analyses in biological processes were performed using the STRING database [14]. A volcano plot was generated using the log2 fold change and adjusted p-values in the DESEq2 results, while whisker plots were prepared for specific genes using the mean log2 normalized counts. Venn diagrams were obtained using Venny 6.1.0 [15] to display the overlap of downregulated genes in siSmyd1 and siPerm1 cardiomyocytes from two sets of RNA-seq data.

### Chromatin-immunoprecipitation (ChIP)-PCR

Heart tissues from 4-month-old WT mice with sham or TAC surgery were cross-linked with formaldehyde. The nuclear fraction was isolated and sonicated to generate a chromatin solution that was then used for immunoprecipitation with anti-Smyd1 antibody (Abcam, # Ab32482). The collected chromatin fragment was validated by qPCR with Maxima SYBR Green qPCR master mix (ThermoFisher, #K0223). Primers used for investigating the rat genome are as follows: (5’ to 3’): *Perm1 Region 1—*CACATTGTCTGAGATCAAGAGTGATGC and CCAGCCCCAAATCTGCCAGCTT; and *Perm1 region 2*—GCCATCCAAACATGACTAACACTGG and CGTTTCCCCTATTTCCAATTCAACTCC.

### Luciferase reporter assays

Luciferase activity was measured with a luciferase assay system (Promega, #E4550). NRVMs were plated on 12-well plates. Reporter plasmids (0.3 μg per well) were transfected with Perm1 expression vector (0.1, 0.3 and 0.7 μg) using Lipofectamine 2000 (Invitrogen, #11668–019) for 24 to 48 hours. Total plasmids were kept at 1 μg-per-well with pDC316 control vector. Harvested cells were lysed with 100 μl Reporter lysis buffer (Promega, #E3971) per well. Luminescence was normalized by protein content. Protein contents were measured by protein assay kit (BioRad, # 5000001). The mean value of the control group (0 μg of Perm1 adenovirus) was expressed as 1. Mammalian expression vector for Perm1 was generated using mouse Perm1 cDNA and pDC316. Reporter plasmids, including ERRE-luc, ERREm-luc, Ndufv1-luc and PGC-1α-luc, were described previously [16, 17].

### Cell Mito Stress Test

Oxygen consumption rates (OCR) in H9c2 cardiomyocytes (ATCC, CRL-1446), were measured using a Seahorse XF^e^96 Analyzer (Agilent) as described previously [2] with some modifications. Briefly, the cells were plated in 96-well Seahorse analyzer plates (2 x 10^4^ cells/well) and transfected with either scrambled-siRNA (Qiagen, #1022076) of siRNA-Perm1 (Qiagen, #SI02021775, #SI02021782). After 48 hours, OCR measurements were conducted periodically, every 4 min over 80 min. Three readings were taken after each injection of oligomycin, carbonyl cyanide-4-(trifluoromethoxy)phenylhydrazone (FCCP), and rotenone + antimycin A. The OCR values were normalized by the intensity of nuclear staining as described previously [18].

### Immunostaining

NRVMs were washed with phosphate buffered saline (PBS) and fixed with 4% paraformaldehyde at room temperature for 10 min. Samples were further washed three times with a PBS solution and permeabilized with a PBS solution containing 0.3% Triton X-100 for 30 min. Then, samples were blocked for 30 min using a PBS solution containing 2% fetal bovine serum (FBS, Sigma Aldrich, #F2442). Samples were, then, incubated overnight at 4°C in a PBS solution containing 2% FBS, anti-cardiac Troponin T (cTnT, Developmental Studies Hybridoma Bank, #RV-C2, 1:50), and anti-Perm1 (Sigma Aldrich, #HPA031712, 1:100). The samples were, then, washed three times with PBS and incubated for 60 min with a goat anti-mouse IgG2b Alexa Fluor^®^ 647 (Invitrogen, #A21241, 1:500), goat anti-rabbit IgG Alexa Fluor^®^ 488 (Invitrogen, #A11034, 1:500) and Hoechst dye (Invitrogen, #H3570, 1:500) diluted in a PBS solution. Following washing with a PBS solution three times, the samples were mounted using ProLong Gold Antifade Reagent (#P36930, Invitrogen). A three-track protocol was used in a Leica SPE confocal microscope (Leica Microsystems, Germany) equipped with a 40x lens. Images were processed using Imaris software (8.4.1 Bitplane AG, Zurich, Switzerland).

### Real-time quantitative polymerase chain reaction (qRT-PCR)

qRT PCR was performed using Taqman primers as described previously [19]. Briefly, RNA was extracted using TRizol (Life Technologies, #15596026), followed by ethanol extraction. Reverse transcription was performed from 1μg of RNA using the QuantiTect Reverse Transcription Kit according to the manufacture’s instruction (Qiagen, #205313). The Cq values from the targeted genes were normalized by the gene expression level of *Tbp*. The following Taqman primers (ThermoFisher) were used: *Tbp* (Cat#: Rn01455646_m1); *Perm1* (Cat#: Rn01749553_g1); *Esrra* (Cat#: Rn00433142_m1); *Esrrg* (Cat#: Rn01415309); *Ppargc1a* (Cat#: Rn00580241_m1); *Ppargc1b* (Cat#: Rn00598552_m1); *PPARa* (Cat#: Rn00566193_m1); *Rxra* (Cat#: Rn00441185_m1); *Nrf1* (Cat#: Rn01455958_m1); *nfe2/2* (Cat#: Rn00582415_m1); *Tfam* (Cat#: Rn00580051_m1); *Hif1a* (Cat#: Rn01472831_m1); *Cs* (Cat#: Rn01774376_g1); *Sdhb* (Cat#: Rn01515728_m1); *Sdha* (Cat#: Rn00590475_m1); *Idh3b* (Cat#: Rn00504589_g1); *Nppa* (Cat#: Rn00664637_g1); *Ndufv1* (Cat#: Rn01454505_m1); *Ndufs1* (Cat#: Rn01438307_m1); *Ndufs8* (Cat#: Rn01450727_g1); *Ndufa8* (Cat#: Rn01438607_m1); *Atp5a* (Cat#: Rn01527025_m1); *Cycs* (Cat#: Rn00820639_g1); *Cpt1b* (Cat#: Rn00682395_m1); *Cpt2* (Cat#: Rn00563995_m1); *Acadm* (Cat#: Rn00566390_m1); *Acox1m* (Cat#: Rn01460628_m1); *Pdhb* (Cat#: Rn01537771_g1); *Slc2a4* (Cat#: Rn00562597_m1); *Mpc1* (Cat#: Rn01524222_g1); *Mpc2* (Cat#: Rn01490486_m1).

### Western blot analysis

Western blot analysis was carried out as previously described [20]. The expression of Perm1, PGC-1α, PGC-1β, ERRα, Smyd1, OXPHOS, SDHB, and UQCRC2 was detected using the following antibodies: HPA031711-100 μl (Sigma); ab54481; ab176328; ab32482, ab76228; ab110411; ab14714; ab14745 (Abcam) diluted 1:1000, followed by goat anti-rabbit secondary antibody (Jackson ImmunoResearch Laboratories, Inc. #711-035-152) or rabbit anti-mouse secondary antibody (Abcam, #ab6728) with 1:5000 dilution. The signal was normalized to either β-tubulin (Abcam, #ab6046) or GAPDH (Cell Singling, #5174).

### Statistical analysis

Data are reported as mean±SEM. Multiple groups were compared by ANOVA, followed by Bonferroni or Tukey post-tests, using GraphPad Prism8 (GraphPad Software, LLC). Unpaired t test was used for direct comparisons between two groups. P<0.05 was considered as statistically significant differences.

## Results

### Perm1 is a downstream target of Smyd1 in cardiomyocytes

**Fig 1:**
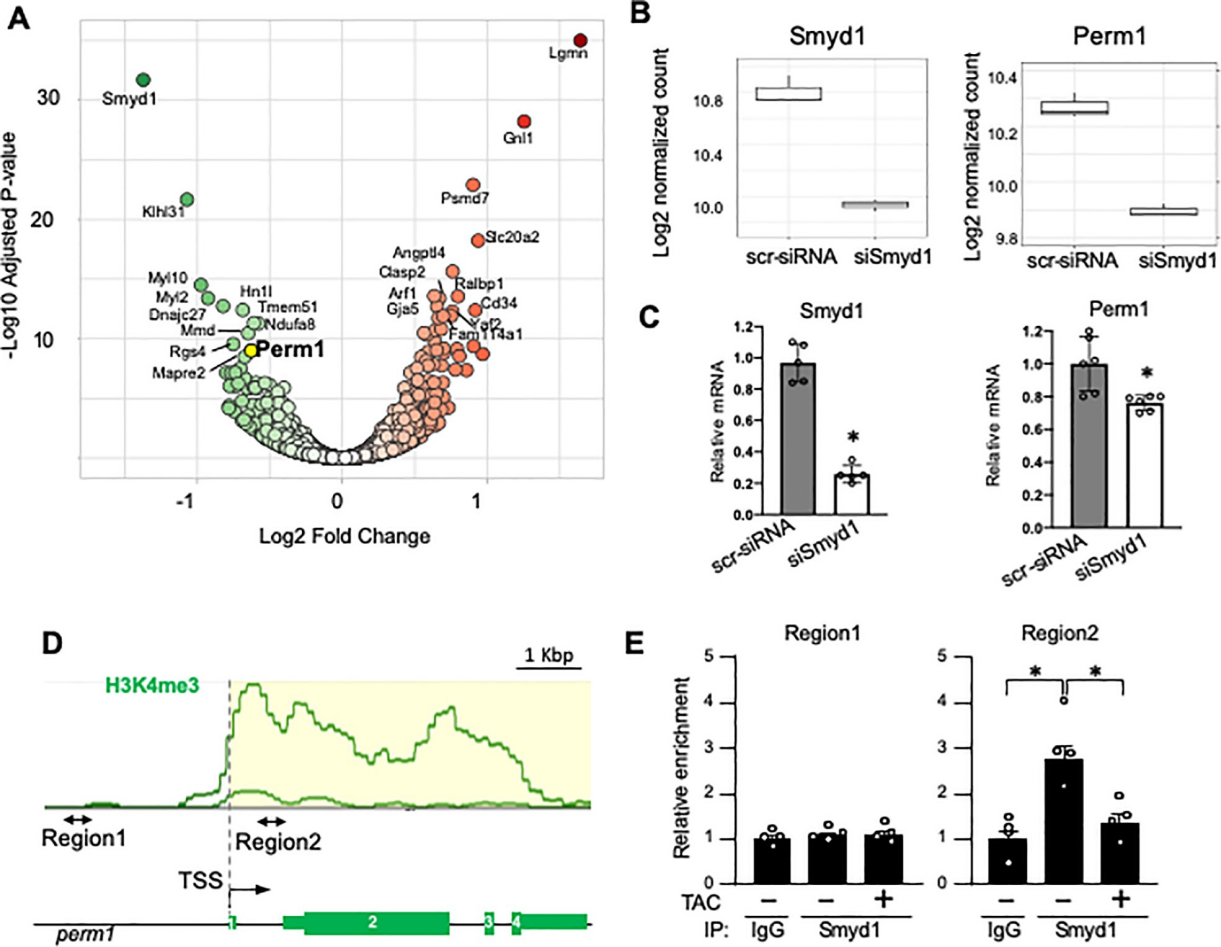
Perm1 is a downstream target of Smyd1. **(A-B)** RNA-seq of siSmyd1 cardiomyocytes. A volcano plot shows significantly downregulated (green) and upregulated (red) genes. Perm1, indicated by a yellow circle, is one of the genes most significantly downregulated by siSmyd1 (Panel A). Box plots show the decreased mRNA levels of Perm1 in cardiomyocytes in which Smyd1 expression was significantly reduced by siSmyd1 as compared with control cells that were treated with scrambled-siRNA (scr-siRNA) (n = 4/group, Panel B). (**C)** qRT-PCR showing a significant reduction in Perm1 in cardiomyocytes in which Smyd1 was knocked-down by siRNA (n = 6/group). Statistics were performed using the t-test with 2 tails. *p<0.05. (**D)** Previous ChIP-seq studies have established enrichment of H3K4me3 within the promoter region of the Perm1 locus (WashU EpiGenome Database), which was targeted for qPCR reaction (“Region 2”). As a negative control, the ~2k bp upstream of the transcription start site (TSS) was targeted (“Region 1”), where small H3K4me3 peaks were observed. (**E)** ChIP-PCR showing no significant interaction of Smyd1 with Region 1 (*left*), while Smyd1 does bind to Region 2 in the hearts of WT mice. This interaction was significantly reduced in the TAC heart (TAC +) (*right*) (n = 4 in sham, n = 4 in TAC). Statistics were performed using the t-test with 2 tails. *: p<0.05. Error bars are ±SEM.

Our previous study showed that the histone methyltransferase Smyd1 regulates cardiac energetics through transcriptionally regulating PGC-1α [2]. However, since adenovirus-mediated overexpression of PGC-1α in cardiomyocytes lacking Smyd1 only partially rescued the reduced mitochondrial respiration caused by Smyd1 deletion [2], it would appear that additional mechanisms exist in Smyd1 regulatory pathways controlling mitochondrial energetics. To seek for Smyd1 target genes that potentially mediate regulation of metabolism, we performed RNA-seq of cultured rat neonatal ventricular myocytes (NRVMs) that had been transfected with either scrambled-siRNA (scr-siRNA, for control) or siRNA-Smyd1 (siSmyd1, for Smyd1 knockdown), as previously performed [2]. RNA silencing reduced *Smyd1* expression to 25.8 ± 2.4% of control (Fig 1C, *left*). We performed STRING database analyses and literature searches on 12 most downregulated and upregulated genes in siSmyd1 NRVMs (Fig 1A) and identified *Perm1* as a potential downstream target of Smyd1 that extends Smyd1 regulatory circuit in cardiac energy metabolism. *Perm1* was significantly downregulated in siSmyd1 cardiomyocytes (1.41 x 10^−9^ in adjusted p-value, Fig 1A and 1B). Real-time quantitative PCR (qRT-PCR) confirmed the reduced mRNA levels of *Perm1* in siSmyd1 cardiomyocytes (76.2 ± 1.9% of control, Fig 1C, *right*).

To investigate whether Smyd1 binds to the promoter of *Perm1*, chromatin-immunoprecipitation (ChIP) assays were performed on ventricular tissue of wild-type (WT) mice through immunoprecipitating using anti-Smyd1 antibody. Smyd1 is known to tri-methylate histone 3 lysine 4 (H3K4me3), which is in general a marker of gene activation [21–23]. Previous ChIP sequencing (ChIP-seq) studies have established the H3K4me3 profile within the Perm1 locus (WashU EpiGenome database, epigenomegateway.wustl.edu), showing the enrichment of H3K4me3 across the entire locus, including a peak at around +500 bases from the transcription start site (TSS) (Fig 1D). Therefore, we examined whether Smyd1 binds to this H3K4me3-rich region of the Perm1 promoter (Region 2). We also examined the binding of Smyd1 to the upstream region (-2 Kb of 5’ region), where small H3K4me3 peaks were observed, as a negative control region (Region 1). No significant interaction of Smyd1 was found in Region 1 (Fig 1E, *left*). In contrast, the Smyd1-immunoprecipitated products were enriched with the DNA from Region 2 (Fig 1E, *right*), suggesting that Smyd1 binds to the TSS region of *Perm1*, presumably to exert transcriptional control. Of note, the interaction of Smyd1 with this H3K4me3-rich region of the *Perm1* promoter (Region 2) was significantly decreased in mouse hearts failing due to pressure overload induced by transverse aortic constriction (TAC +) (Fig 1E, *right*), suggesting that epigenetic modifications occur on the *Perm1* locus during hemodynamic stress. Using DNA binding assays, we were unable to detect the direct binding of Smyd1 to H3K4me3-rich region of *Perm1* promoter (S1 Text and S1 Fig). Given that Smyd1 is a histone modifier and it is currenly believed that Smyd1 does not directly bind to DNA [23], it is likely that the affinity of Smyd1 to the DNA without histone may be limited. Further investigation is needed to clarify how the Smyd1 interaction with the Region 2 of the *Perm1* promoter is mediated. In summary, our data suggest that *Perm1* is a target gene of Smyd1 in cardiomyocytes.

### Perm1 is downregulated in the failing heart in association with the release of Smyd1 from its promoter

**Fig 2:**
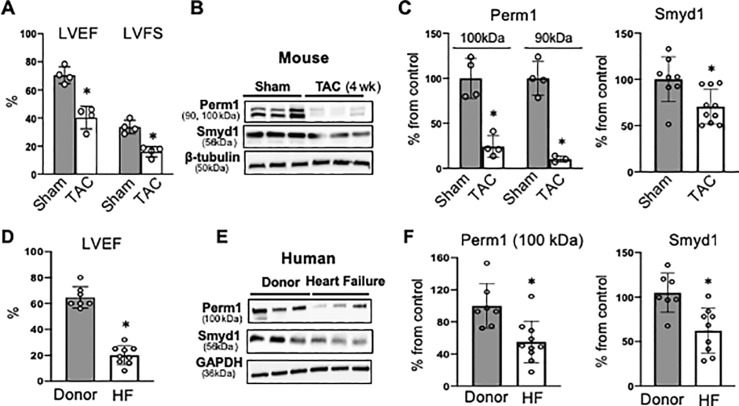
Perm1 is downregulated in the failing heart. **(A-C)** Downregulation of Perm1 and Smyd1 in mouse hearts subjected to TAC surgery for 4 weeks, where left ventricular ejection fraction (LVEF) and fractional shortening (LVFS) were significantly decreased as compared with sham-operated hearts (n = 4/group for Perm1 expression, n = 8-10/group for Smyd1 expression) (also see S1 Table). Consistent with previous studies in the mouse skeletal muscle [10, 25, 26], Western blotting analysis shows two isoforms of Perm1 at 90 kDa and 100 kDa in the mouse heart (*Panel B*). (**D-F)** Downregulation of Perm1 and Smyd1 in patients with heart failure, where LVEF was significantly decreases as compared with donor hearts (n = 7 in donor group, n = 9–10 in heart failure patients, also see S2 Table). Statistics were performed using the t-test with 2 tails. *: p<0.05. Error bars are ±SEM.

Given that H3K4me3 generally leads to activation of genes [24] and that Smyd1 dissociates from the H3K4me3-rich promoter region of the Perm1 locus in response to pressure overload (Fig 1E), we hypothesized that Perm1 is downregulated in the failing heart. To test this hypothesis, we measured the protein levels of Perm1 in the mouse and human failing hearts. Consistent with a previously study, we detected two isoforms of Perm1 in the mouse heart, at 100 kDa and 90 kDa, in the mouse heart (Fig 2B). At 4 weeks post-TAC, ejection fraction and fractional shortening were both significantly decreased in mluse hearts (Fig 2A, S1 Table), and the protein levels of the 100 kDa and 90 kDa Perm1 isoforms were decreased to 24.4 ± 5.9% and 10.3 ± 2.0%, respectively, compared to in the sham heart (Fig 2B and 2C). Similarly, Perm1 was significantly decreased in cardiac tissue that was collected from heart failure patients at the time of implantation of a left ventricular assist device (55.2 ± 13.1% of donors, Fig 2E and 2F) compared to in tissue from donor hearts (see Fig 2D and S2 Table for cardiac function and donor and patient information). The dispersion of Perm1 expression was relatively large in the failing hearts. Due to the uneven distribution of male and female patients in this dataset (n = 8 in male and n = 2 in female), it is not possible to ascertain at this point whether downregulation of Perm1 has gender specificity. Of note, we detected only one isoform at 100 kDa in the human hearts, except in one donor heart that showed a faint band around 90 kDa (Fig 2E). It remains unclear whether the 100 kDa protein is a dominant form of Perm1 in human. We also found that Smyd1 was downregulated in those TAC mouse hearts (70.5 ± 5.9% of sham, p<0.05, Fig 2C, right) and human failing hearts (62.2 ± 8.5% of donor, p<0.05, Fig 2F, right). Thus, our data showed that Perm1 is downregulated in the failing heart, which could be attributed to a combination of two phenomena, the release of Smyd1 from the H3K4me3-rich promoter region of the *Perm1* gene as well as decreased expression levels of Smyd1 in the heart.

### Perm1 knockdown reduces mitochondrial energetics in cardiomyocytes

**Fig 3:**
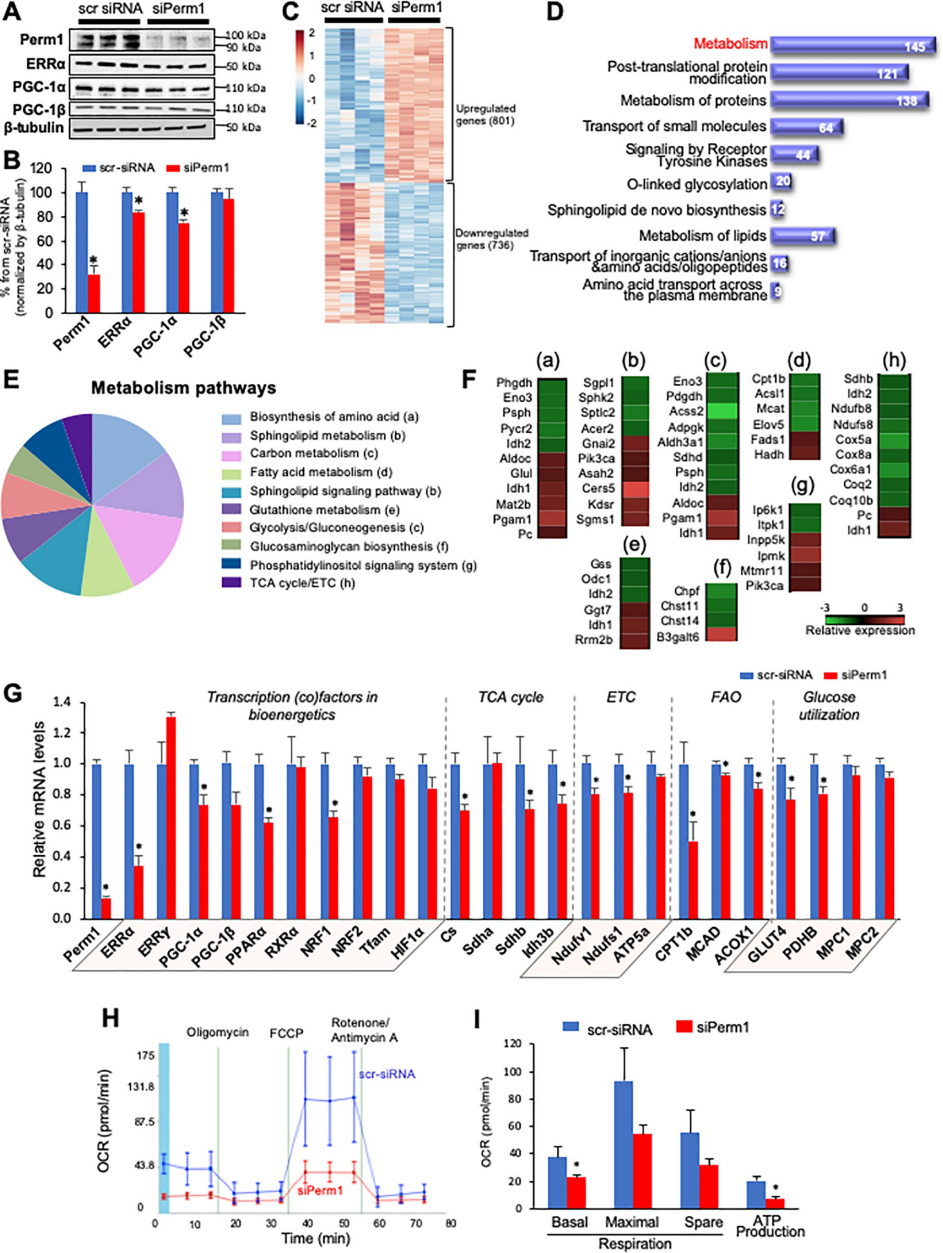
Perm1 positively regulates metabolism in cardiomyocytes. **(A-B)** Western blot analysis showing a significant reduction in the endogenous protein levels of Perm1, ERRα, and PGC-1α in NRVMs that were transfected with siPerm1, as compared with control cells that were treated with scrambled-siRNA (scr-siRNA) (n = 4/group). (**C-F)** Illumina-based RNA-seq showing that differentially expressed genes in siPerm1 NRVMs as compared with control include 801 upregulated genes and 736 downregulated genes (n = 4/group, p<0.05, *Panel C*, also see S2 Dataset). Enrichment analysis of significantly regulated genes for the Reactome terms biological pathways shows that the pathway most affected by Perm1 knockout is metabolism (*Panel D*, S3 Dataset). Pie charts representing the major metabolic pathways of differentially regulated genes involved in metabolism in siPerm1 cardiomyocytes (*Panel E*, S4 Dataset). Heat maps of genes comprising the pie chart presented in Panel E display the dictionary of changes by Perm1 knockout (*Panel F*). (a) Biosynthesis of amino acid, (b) Sphingolipid metabolism and signaling pathway, (c) Carbon metabolism and glycolysis, (d) Fatty acid metabolism, (e) Glutathione metabolism, (f) Glucosaminoglycan biosynthesis, (g) Phosphatidylinositol signaling system, (h) TCA cycle / electron transport chain (ETC). (**G)** qRT-PCR showing the expression of genes involved in energetics in Perm1-knockdown (siPerm1) led to downregulation of energetics-related genes as compared to in control cells (n = 3-5/group, ±SEM). (**H)** Cell Mito Stress Test was performed using a Seahorse XF96 analyzer (n = 8/group, ±SD). O_2_ consumption rate (OCR) was measured in the presence of glucose as a major respiration substrate. **(I)** Results were normalized by nuclear staining as described in our publication [2]. Error bars are ±SEM. Statistics were performed using the t-test with 2 tails. *p<0.05.

The effect of *Perm1* downregulation in cardiac muscle is unknown. To determine the genes and biological pathways that are regulated by *Perm1*, we performed RNA-sequence (RNA-seq) analysis of NRVMs, which were transfected with either scr-siRNA (for control) or siRNA-Perm1 (siPerm1, for Perm1 knockdown). siPerm1 decreased the protein expression level of Perm1 at 100 kDa to 32.0 ± 6.7% of control cardiomyocytes (scr-siRNA). We were unable to quantify the degree of suppression of the 90 kDa Perm1 isoform due to the bands being undetectable in the siPerm1 group (Fig 3A). Western blotting analysis also showed downregulation of ERRα and PGC-1α by Perm1 knockdown (both p<0.05), while PGC-1β expression was not affected (Fig 3A and 3B). Illumina-based RNA-seq analysis of control (scr-siRNA) and siPerm1 cardiomyocytes detected 15,032 genes, among which 1,537 genes were significantly altered by *Perm1* knockdown (801 upregulated genes, 736 downregulated genes, p<0.05, Fig 3C, S2 Dataset). Using the STRING Database web-based tools [27], the differentially expressed genes in siPerm1 cardiomyocytes were analyzed for biological pathways based on the Reactome database (S3 Dataset). The 10 most significantly enriched biological pathways are shown in Fig 3D. The pathway most affected by *Perm1* knockdown was metabolism (145 genes). To further explore the role of Perm1 in metabolism, we analyzed those genes for Kyoto Encyclopedia of Genes and Genomes (KEGG) terms, revealing that *Perm1* regulates various metabolic pathways, including biosynthesis of amino acids, sphingolipid metabolism, carbon metabolism, fatty acid metabolism, glutathione metabolism, glycolysis, glycosaminoglycan biosynthesis, the phosphatidylinositol signaling system, and OXPHOS (the TCA cycle and electron transport chain) (Fig 3E, S4 Dataset). The comparative analysis of genes in those pathways is depicted in heat maps (Fig 3F), displaying the direction of differentiated expression compared with control. This analysis of the subset of genes involved in metabolism highlights downregulation of key enzymes/transporters in energetics, such as the tricarboxylic acid (TCA) cycle enzymes (*Sdhb; Idh2*), the subunits of protein complexes comprising the electron transport chain (ETC) (*Ndufb8; Ndufs8; Cox5a; Cox8a; Cox6a1; Coq2; Coq10b*), the transporter and enzyme of fatty acid β-oxidation (FAO) (*Cpt1b; Acsl1*), and the glycolytic enzymes (*Eno3; Pdgdh*) (Fig 3F). In contrast, the genes involved in biosynthesis of amino acids (*Psph; Pycr2*), sphingolipid metabolism (*Gnai2; Pik3ca; Asah2; Cers5; Kdsr; Sgms1*), and the phosphatidylinositol signaling system (*Inpp5k; Ipmk; Mtmr11; Pik3ca*) were upregulated by *Perm1* knockdown (Fig 3F).

Consistent with the RNA-seq data, qRT-PCR showed that Perm1 knockdown in cardiomyocytes led to downregulation of genes encoding for the ETC subunits (*Ndufv1; Ndufs1*), the TCA cycle (*Cs; Sdh3b; Idh3b*), and enzymes and transporters involved in FAO (*Cpt1b; MCAD; Acox1*) and glucose utilization (*Gult4; Pdhb*) (all p<0.05, Fig 3G). *Perm1* knockdown also led to downregulation of the transcription (co)factors that coordinate gene expression for energetics, including *ERR*α, *PGC-1α*, peroxisome proliferator-activating receptor alpha (*PPARα*, a transcription factor that regulates FAO), and nuclear receptor factor 1 (*NRF1*, a transcription factor that regulates mitochondrial bioenergetics) (all p<0.05, Fig 3G). Among these, the effect of *Perm1* knockdown on expression of *ERR*α, a transcription factor that regulates mitochondrial bioenergetics, was striking. The mRNA level of *ERR*α was decreased to 34.1 ± 6.8% of control in siPerm1 cardiomyocytes (p<0.05, Fig 3G). In contrast, the expression level of *ERR*γ, an isoform of *ERRα*, was not affected by *Perm1* knockdown (Fig 3G). We also did not find any significant changes in the expression of mitochondrial transcription factor A (*Tfam*), a regulator of mitochondrial biogenesis, or hypoxia inducible factor 1-alpha (*Hif1α*), a regulator of glycolytic genes (Fig 3G). Overall, our data showed that knockdown of *Perm1* leads to global downregulation of genes involved in OXPHOS.

To determine if Perm1 regulates mitochondrial respiration capacity, we performed the Cell Mito Stress Test in H9c2 cardiomyocytes using a Seahorse 96XF flux analyzer. Perm1 knockdown significantly reduced the basal respiration rate and ATP production as compared with control cells (40.7% and 23.6% of scr-siRNA, respectively, both p<0.05, Fig 3H and 3I). These data suggest that Perm1 is required to maintain mitochondrial energetics in cardiomyocytes.

### Perm1 induces metabolic genes in cardiomyocytes

**Fig 4:**
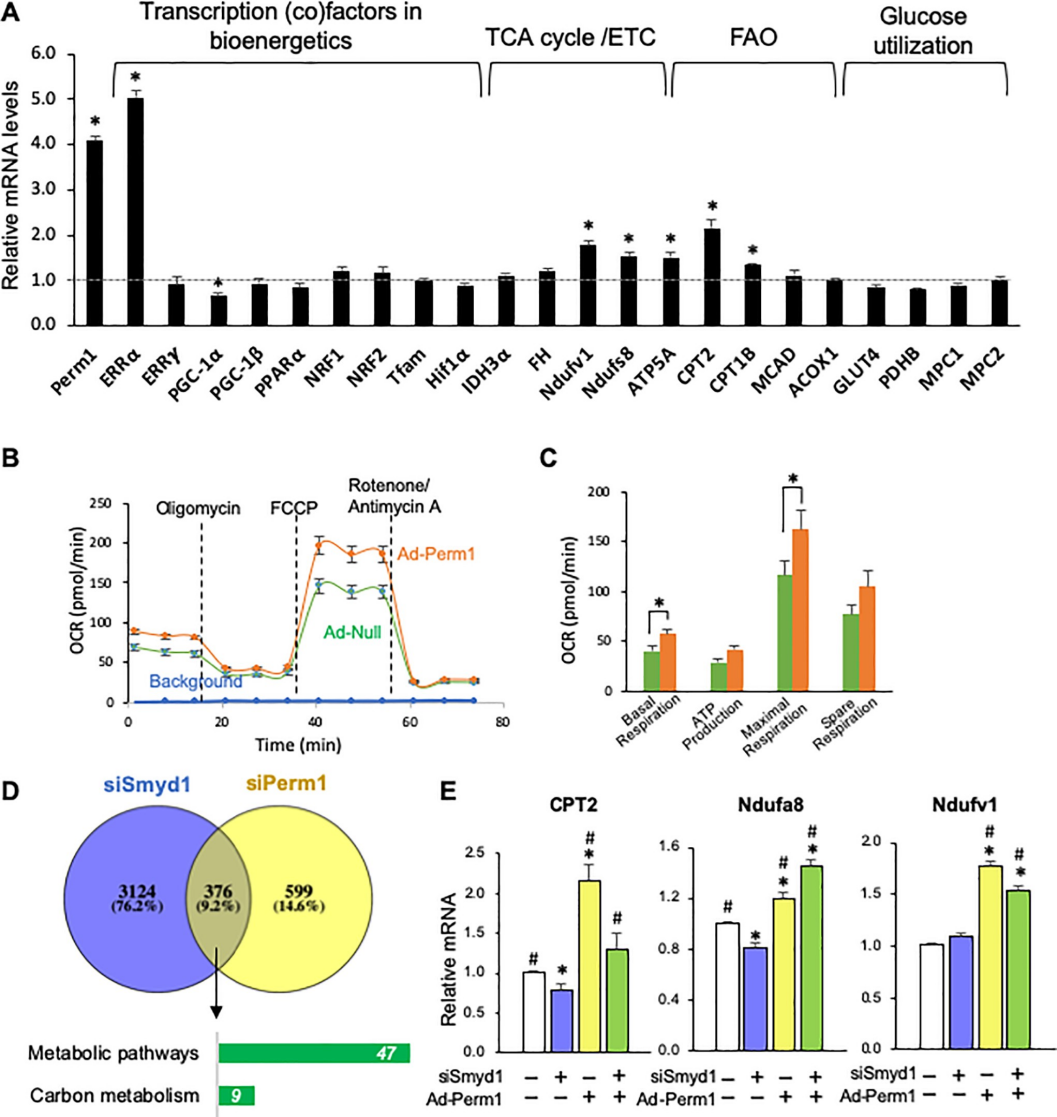
Adenovirus-mediated overexpression of Perm1 (Ad-Perm1) enhances energetics in cardiomyocytes, partially mediated by Smyd1. **(A)** qRT-PCR showing the expression of genes involved in energetics (n = 4/group). Statistics were performed using the t-test with 2 tails. *p<0.05. (**B)** Cell Mito Stress Test was performed using a Seahorse XF96 analyzer in NRVMs (n = 8/group, ±SD). O_2_ consumption rate (OCR) was measured in the presence of glucose as a major respiration substrate. **(C)** Results were normalized by nuclear staining as described in our publication [2]. Error bars are ±SEM. Statistics were performed using the t-test with 2 tails. *p<0.05, (**D)** A Venn diagram showing the genes downregulated in both siSmyd1 and siPerm1 NRVMs that were detected in RNA-seq (top). The overlaping genes were further analyzed by KEGG pathway analysis (bottom). (**E)** qRT-PCR shows that siSmyd1 downregulated *CPT2* and *Ndufa8*, both of which were completely rescued by Perm1overexpression (Ad-Perm1). In contrast, Ndufv1 expression was not affected by siSmyd1, suggesting that Ndufv1 is not a Smyd1 target gene. Ndufv1 was upregulated by Ad-Perm1 in the presene and absence of siSmyd1 (n = 3-5/group, t-test). *: p<0.05 compared with control, #: p<0.05 compared with siSmyd1. Statistics were performed using one-way ANOVA. Error bars are ±SEM.

To test whether Perm1 can induce metabolic genes, *Perm1* was transduced into NRVMs using adenovirus (Ad-Perm1, for 48h). The *Perm1* mRNA level in Ad-Perm1 cardiomyocytes was increased 4 fold, as compared with control cardiomyocytes that were treated with null adenovirus (Ad-null) (p<0.05) (Fig 4A). Consistent with the effect of *Perm1* knockdown (Fig 3G), *Perm1* overexpression led to remarkable upregulation of *ERR*α, whereas *ERR*γ expression was slightly increased (1.31 in fold change, however, this difference was not statistically significant (p = 0.25) (Fig 4A). The increased expression of *Perm1* and *ERR*α was accompanied by upregulation of *Ndufv1*, *Ndufs8* (Complex I), *ATP5a* (Complex VI), *CPT1b*, and *CPT2* (FAO) (all p<0.05, Fig 4A). In contrast, the expression of genes involved in glucose metabolism (*Hif1α*, *Glut4*, *Pdhb*, *Mpc1*, and *Mpc2*) did not change. Of note, the *PGC1*α mRNA level was reduced by *Perm1* overexpression (66.9 ± 7.3% of control, p<0.05). These data suggest that *Perm1* induces *ERR*α and a subset of OXPHOS genes.

To determine whether the upregulation of ERRα and some OXPHOS genes by Ad-Perm1 enhances mitochondrial energetics, we performed a Cell Stress Test using those cardiomyocytes. Basal and maximal respiration capacity was significantly increased by Perm1 overexpression (144% and 140% of Ad-null, respectively, both p<0.05, Fig 4B and 4C).

To investigate whether the metabolic genes regulated by Perm1 are downstream targets of Smyd1, we performed a bioinformatic analysis of genes downregulated by siSmyd1 and siPerm1, obtained from RNA-seq (Fig 1A and 1B and Fig 3C–3F, S1 and S2 Datasets). Using Venny 6.1.0. software (Centro Nacional De Biotechnoligia (CSIC), Spain), we generated a Venn diagram as shown in Fig 4B. We found 376 shared genes that were downregulated in both the siSmyd1 and siPerm1 groups, which were further analyzed for biological pathways using the STRING Database web-based tools. Only two enriched downregulated KEGG term pathways were found: metabolic pathways and carbon metabolism (Fig 4D, bottom, S5 Dataset), suggesting that Smyd1 regulates some metabolic genes through Perm1. Pathways upregulated in both siSmyd1 and siPerm1 cardiomyocytes include regulation of actin cytoskeleton and the sphingolipid signaling pathway (S6 Dataset). We further tested whether *Perm1* overexpression can rescue downregulated metabolic genes in siSmyd1 cardiomyocytes. NRVMs were transfected with siSmyd1, followed by transduction of *Perm1* using adenovirus. qRT-PCR showed that carnitine palmitoyltransferase 2 (*CPT2*), which is an important enzyme in FAO, was significantly downregulated in siSmyd1 cardiomyocytes (79 ± 8% of control, p<0.05), but was completely rescued by *Perm1* overexpression (131 ± 8% of control, p>0.05 as compared with control, p<0.05 as compared with siSmyd1, Fig 4E). Of note, adenovirus-mediated overexpression of *Perm1* itself increased the mRNA level of *CPT2* by 2.2 fold (p<0.05 as compared with control and siSmyd1 groups). Similarly, the mRNA level of *Ndufa8* (Complex 1) was significantly decreased by *Smyd1* knockdown (81 ± 3% of control, p<0.05 as compared with control), but was increased to 146 ± 5% by *Perm1* overexpression (p<0.05 as compared to control and siSmyd1, Fig 4E), although the increase in the mRNA level of *Ndufa8* by Ad-Perm1 was relatively small (1.2 in fold change, p<0.05 as compared with control and siSmyd1 groups). On the other hand, the expression level of *Ndufv1* (Complex 1) was not changed by *Smyd1* knockdown (109 ± 8% of control, p>0.05 as compared to control), suggesting that this component is not a Smyd1 target gene. *Perm1* overexpression increased the mRNA level of *Ndufv1* (177 ± 12% of control, p<0.05), and it remained upregulated in siSmyd1 + Ad-Perm1 cardiomyocytes (154 ± 20% of control, p<0.05 as compared with control and siSmyd1, Fig 4E). Together with downregulation of this gene by siPerm1 (Fig 3G), our data suggest that *Ndufv1* gene expression is not regulated by Smyd1, but rather it is a direct target of Perm1. Overall, these data suggest that Perm1 positively regulates genes involved in energetics, including, but not limited to, a subset of Smyd1 target genes.

### Perm1 rescues downregulation of metabolic genes during hypertrophic stress in cardiomyocytes

**Fig 5:**
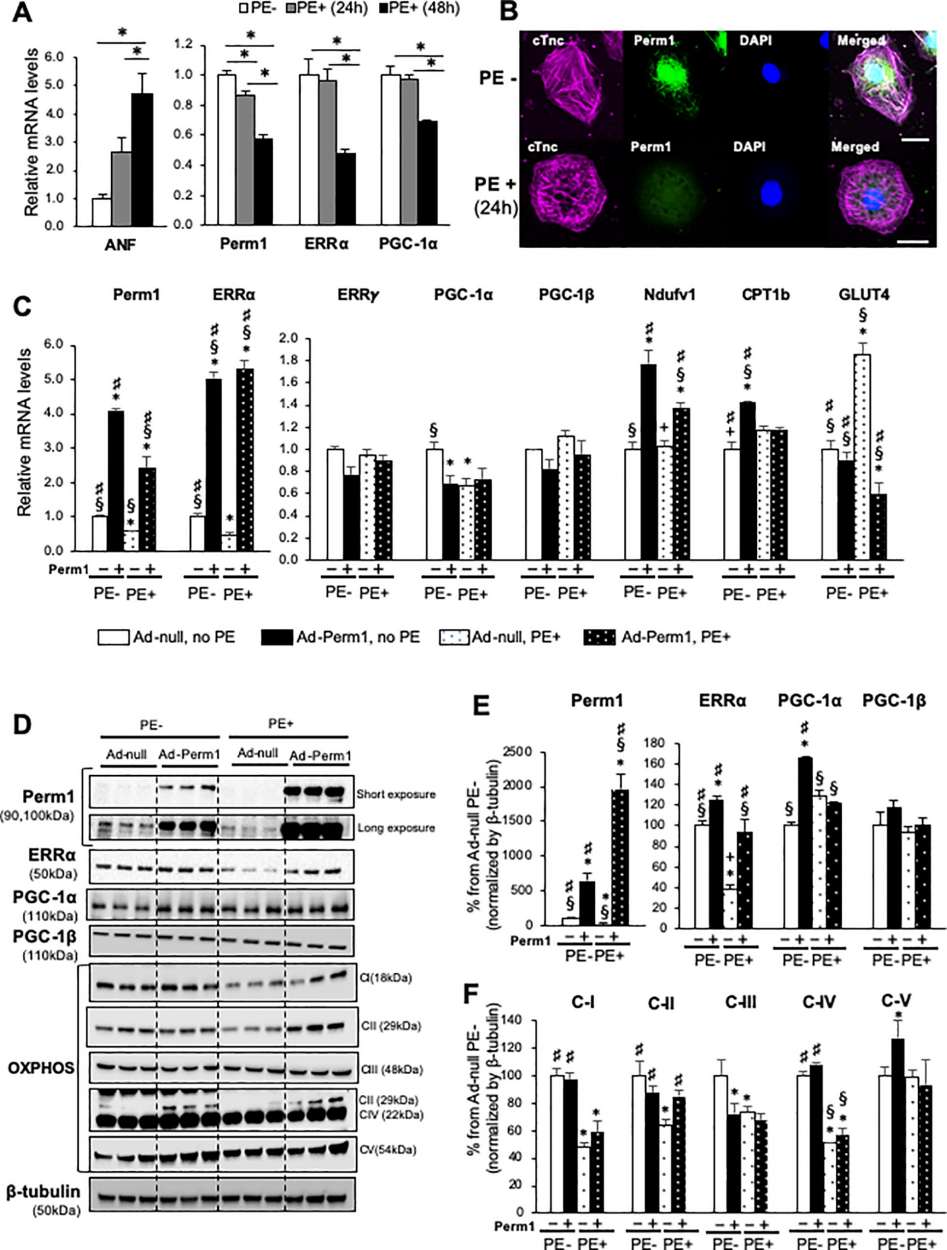
Perm1 is downregulated in cardiomyocytes under cellular hypertrophic stress. **(A)** RT-PCR showing Perm1 downregulation in NRVMs treated with phenylephrine (PE) (n = 3/group *:p<0.05 by one-way ANOVA). (**B)** Immunostaining of NRVMs showing the localization of Perm1 in the nucleus and the peripheral nuclear region (Top). After incubation with PE for 24hr, Perm1 was less localized in the nucleus and showed a diffuse expression pattern. Cardiac muscle-specific anti-troponinC (cTnc) was used to distinguish cardiomyocytes from fibroblasts. Scale bar: 20 μm. (n = 3/group). (**C-F)** RT-PCR (*Panel C*) and Western blot analysis (*Panels D-F*) showing that Perm1 overexpression prior to 48h incubation with PE (group Perm1+/ PE+) either completely or partially rescues downregulation of some metabolic genes during PE-induced hypertrophic stress. The control (group Perm1-/PE-) was obtained by treating NRVMs with adenovirus-null for 48h without incubating with PE. The data for group Perm1-/PE- and group Perm1+/PE- in Panel C is the same as shown in Fig 4A. Note that the expression levels of the Complex II and III subunits were quantified using individual antibodies againt SDHB and UQCRC2 in separate blots due to being adjacent to the band from the relatively highly abundant Complex IV subunit. Statistics were performed using one-way ANOVA. *: p<0.05 compared with Perm1-/PE- (control); §: p<0.05 compared with Perm1+/PE+; #: p<0.05 compared with Perm1-/PE-. Error bars are ±SEM.

Treatment of NRVMs in vitro with phenylephrine (PE), an α1-adrenergic agonist, causes a repression of energetics-related genes, recapitulating metabolic remodeling in response to pressure overload [28]. To investigate wheter PE-induced hypertrophic stress downregulates Perm1in a manner similar to what we observed in the TAC heart (Fig 2B and 2C), NRVMs were treated with 50 μM PE for 24 and 48h, respectively. qRT-PCR showed that *Perm1* expression was already significantly decreased after the 24h incubation with PE, and was further decreased after the 48h incubation, concurrent with a progressive increase in the expression of atrial natriuretic factor (*ANF*), a marker of cellular hypertrophy (Fig 5A). We also observed a significant decrease in the mRNA levels of *ERR*α and *PGC-1α* after the 48hr incubation with PE, but there was no change at 24h (Fig 5A), suggesting that *Perm1* downregulation occurred prior to downregulation of ERRα and *PGC-1α*.

Immunostaining analysis of NRVMs showed that Perm1 was predominantly localized in the nuclear and pre-nuclear regions of troponin C (cTnc)-positive cells (Fig 5B, top). Consistent with the results in qRT-PCR results, the Perm1 signal was reduced, with a diffused pattern, after the incubation with PE for 24h (Fig 5B, bottom).

PE incubation initially led to downregulation of Perm1, followed by a significant reduction in mRNA levels of the metabolic regulators *ERR*α and *PGC-1α* (Fig 5A and 5B). These data suggest that the repression of those genes by PE may be, in part, mediated by downregulation of *Perm1* itself. We examined the ability of Perm1 to reverse the effects of PE by introducing exogenous *Perm1* via adenoviral delivery, as performed in Fig 4, prior to the incubation of PE. RT-PCR and Western blotting analysis consistently showed that *Perm1* overexpression fully prevented the PE-induced downregulation of ERRα (Fig5C–5E). In contrast, the protein level of PGC-1α was not significantly changed by PE. Note that Ad-Perm1 increased the protein expression level of PGC-1α (Fig 5E, p<0.05), although its mRNA level was decreased by Ad-Perm1 (Fig 5C). The expression of neither ERRγ nor PGC-1β was changed by PE or *Perm1* overexpression (Fig 5C–5E). Western blotting analysis showed that PE incubation decreased the expression of Complex I, II, and III, and IV; among these, downregulation of Complex II was rescued by Perm1 overexpression (Fig 5D and 5F). Interestingly, *GLUT4* was upregulated by PE incubation, an effect that was completely abolished by *Perm1* overexpression (Fig 5C). Overall, our data suggest that *Perm1* has the ability to restore the expression of a subset of genes involved in mitochondrial energetics during PE-induced hypertrophic stress.

### Perm1 directly regulates OXPHOS genes through the ERRE

**Fig 6:**
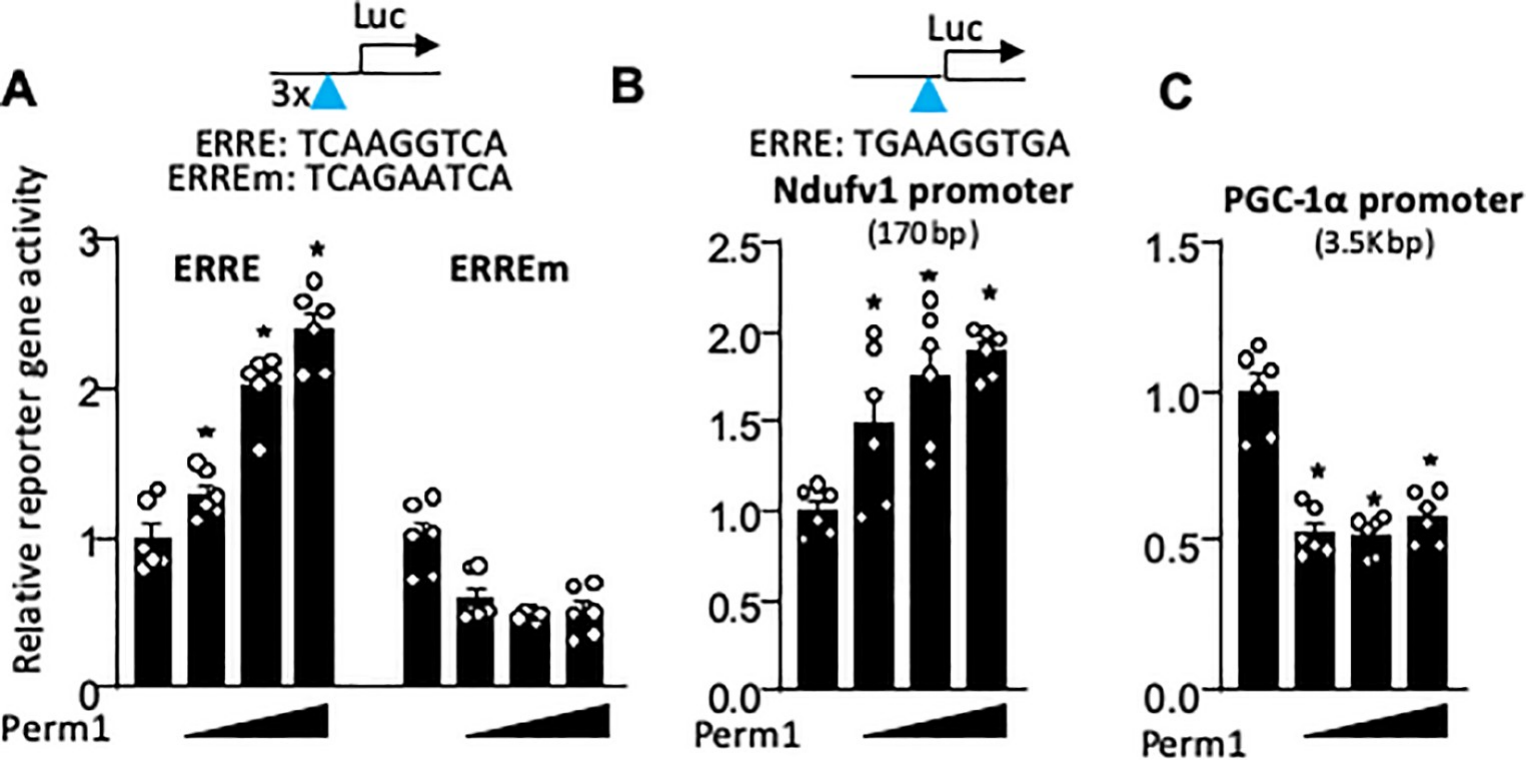
Perm1 activates the promoters of ERRα and its target gene through the ERRE. (A) Luciferase reporter assays showing that Perm1 increases ERRE activity (TCAAGGTCA) in a dose-dependent manner, whereas mutant ERRE (mERRE, TCAGAATCA) is not be activated by Perm1. (B) Perm1 activates the ERRE within the *Ndufv1* promoter (a subunit of Complex I, an ERRα target gene) in a dose-dependent manner. Note that the ERRE in mouse *Ndufv1* promoter is TGAAGGTGA. (C) Perm1 does not increase the promoter activity of *PGC-1α* but rather represses the gene (n = 5/group). Statistics were performed using the t-test with 2 tails (*p<0.05). Error bars are ±SEM.

The qRT-PCR data presented above in Fig 3G and Fig 4A reveal the concordant changes in the expression of ERRα and its target gene *Ndufv1* in response to manipulations of *Perm1* expression: both these genes are significantly downregulated by *Perm1* silencing and are significantly upregulated by *Perm1* overexpression. ERRα target genes contain a specific DNA sequence in their promoter regions known as an ERR-alpha response element (ERRE), which is critical for transcription control. *ERR*α itself also includes an ERRE in its promoter region, which allows feedback to boost transcription in response to increased energy demand. We hypothesized that Perm1 transcriptionally regulates *ERR*α and ERRα target genes involved in OXPHOS through the ERRE. To test this hypothesis, we performed luciferase reporter gene assays in NRVMs. We found that Perm1 increases the promoter activity driven by three repeats of the ERREs (TCAAGGTCA) in a dose-dependent manner, while the mutant ERRE (TCAGAATCA) was not activated by Perm1 (Fig 6A). Similarly, Perm1 increased the activity of the *Ndufv1* promoter containing an ERRE, in a dose-dependent manner (Fig 6B). In contrast, Perm1 failed to increase the promoter activity of *PGC-1α* but rather suppressed it. This is consistent with qRT-PCR data showing that *Perm1* overexpression significantly decreased *PGC-1α* mRNA in *NRVMs* (Fig 4A). In summary, these data suggest that Perm1 transcriptionally activates *ERR*α and its target genes through the ERRE.

## Discussion

We previously identified the histone methyltransferase Smyd1 as a regulator of mitochondrial energetics in the heart. Smyd1 is a muscle-specific epigenetic regulator that tri-methylates histone 3 lysine 4 (H3K4me3), a marker of gene activation [2]. Smyd1 ablation in the adult mouse heart leads to global downregulation of OXPHOS proteins, concurrent with downregulation of PGC-1α, a key regulator of mitochondrial energetics, and decreased levels of H3K4me3 on the *PGC-1α* promoter, which allowed us to conclude that Smyd1 regulates cardiac energetics through transcriptional control of *PGC-1α* [2]. Herein, we demonstrate that Perm1 is another downstream target of Smyd1 in metabolic networks. Similar to Smyd1, Perm1 is predominately expressed in the skeletal and cardiac muscle, suggesting that Perm1 mediates muscle-specific regulatory mechanisms in response to increased energy demands.

We found that Perm1 is downregulated in the mouse and human failing hearts, as wells as in cardiomyocytes in response to cellular hypertrophic stimuli. Downregulation of Perm1 in failing hearts are correlated with two phenomena: the decreased expression of Smyd1 (Fig 2) and the release of Smyd1 from the H3K4me3-rich promoter region of *Perm1* (Fig 1E). Of interest, it was previously reported that the protein level of chromatin-bound Smyd1 is increased in mouse hearts subjected to pressure overload [20]. Unfortunately, that report did not indicate the duration of TAC when the measurements were taken, and did not present a parallel measurement of the global Smyd1 expression in the heart. It is therefore possible that chromatin-bound fraction of Smyd1 increases in response to TAC *despite* global decrease in Smyd1 expression. Another possibility in chromatin-bound Smyd1 is a transient phenomenon occurring early in the heart failure development, which is reverted in more mature stages of the disease. Perhaps a detailed parallel analysis of whole-cell and chromatin-bound Smyd1 during different time points of pressure overload-induced heart failure develppment should be performed in a future study.

It also remains unclear what causes the reduced interaction of Smyd1 with the *Perm1* promoter region under pressure overload (Fig 1E). We were unable to detect binding of Smyd1 to biotin-labeled DNA containing the H3K4me3-rich region (S1 Fig), which confrimes the current opinion tha Smyd1 does not directly bind to DNA [23]. Given that Smyd1 is known to be a histone modulator and can regulate gene expression through chromatin remodeling [21], it is possible that chromatin is required for Smyd1 to interact with the *Perm1* promoter. Summarizing, our data suggests that the Perm1 gene undergoes epigenetic modifications under pressure overload, which is correlated with the decrased presence of Smyd1 at the *Perm1* promoter. However, the overall signaling vector from Smyd1 and Perm1 during evolving heart failure appears to be highly complex and needs further calrification. It remains to be demonstrated whether the release of Smyd1 from the *Perm1* promoter is a necessary or sufficient factor of Perm1 downregualtion in heart failure.

We further demonstrated that Perm1 is an essential regulator of mitochondrial function and substrate metabolism in cardiomyocytes. Decreased availability of endogenous Perm1 in primary cardiomyocytes led to downregulation of various metabolic genes encoding for the enzymes and components of the TCA cycle, the ETC, FAO, and glucose utilization, which was concurrent with a reduced capacity for mitochondrial repair. These results are consistent with previous observations in C2C12 myoblasts, which showed that Perm1 regulates a subset of PGC-1s/ERRs target genes [10]. Additionally, our RNA-seq data obtained from cardiomyocytes in which *Perm1* was knocked-down by siRNA suggest that Perm1 broadly regulates metabolism, including glycolysis, amino acid metabolism, and sphingolipid signaling pathways. Thus, it is possible that Perm1 not only regulates mitochondrial energetics but is also involved in cellular processes and homeostasis.

Our study shows that phenylephrine-induced hypertrophic stress, which recapitulates metabolic remodeling under pressure overload, leads to downregulation of *Perm1*, followed by downregulation of *ERR*α and *PGC-1α* (Fig 5A). It is possible that downregulation of Perm1 might occur during the early stage of heart failure under hemodynamic stress, leading to dysregulation of ERRα/PGC-1α signaling in mitochondrial bioenergetics. It is of interest to determine whether maintaining Perm1 expression during pressure overload can prevent energetic defects. In support of this notion, our study showed that adenovirus-mediated overexpression of *Perm1* rescues downregulated *ERR*α and some OXPHOS genes during hypertrophic stress in cardiomyocytes.

Perm1 was discovered by Cho and colleagues as a protein induced by adenovirus-mediated overexpression of *PGC-1α*, *PGC-1β*, *ERRβ*, and *ERR*γ in differentiated C2C12 cells [10]. Hence, it was defined as “PGC-1- and ERR-induced regulator, muscle 1” [10]. In that original study, gain-of-function of Perm1 did not alter the expression of either *PGC*-1α or *ERR*α, indicating a “linear” signaling from the PGC-1α/ERRα complex to Perm1. However, a subsequent study from the same group showed that AAV-mediated *Perm1* overexpression in mouse skeletal muscle significantly increased the expression of both *ERR*α and *PGC-1α*, suggesting a feed-forward regulatory loop formed by PGC-1α/ERRα and Perm1 [25]. In our study, both loss-of-function and gain-of-function of Perm1 in cardiomyocytes prominently altered *ERR*α expression, enforcing the notion that Perm1 targets ERRα and acts as an upstream regulator of ERRα. In contrast, *PGC-1α* expression was reduced by both loss-of-function and gain-of-function of Perm1 at the mRNA levels.

The exact molecular mechanism by which Perm1 regulates expression of OXPHOS genes is currently unknown. Previous studies in mouse skeletal muscle suggested that Perm1 regulates OXPHOS genes through activation of p38 mitogen activated protein kinase (p38MAPK) and Ca2+/calmodulin-dependent protein kinase II (CaMKIIβ) [Cho 2016, Cho 2019]. Exercise-induced activation of p38MAPK not only directly activates PGC-1α through phosphorylation [29] but also phosphorylates two well-known transcription factors, myocyte enhancer factor 2 (MEF2) [30] and activating transcription factor 2 (ATF2) [31], to increase transcription of the *PGC-1α* gene, thereby creating an autoregulatory feed forward loop by which PGC-1α increases its own expression [32]. CaMKIIβ, the main CaMK isoform in skeletal muscle, is upstream of p38MAPK [33]; thus, activation of CaMKIIβ can induce PGC-1α through p38MAPK [34]. Given that PGC-1α can also increase *ERR*α expression [35], upregulation of *ERR*α in AAV-*Perm1* expressing skeletal muscle may be explained by the increased expression of *PGC-1α*.

We currently have no information as to whether Perm1 activates p38 or CaMKII in cardiomyocytes. However, our study suggests a different mechanism of Perm1 signaling, which is associated with the ERR-response element (ERRE) [36]. The ERRE contains a single consensus half-site, 5’-TNAAGGTCA-3’. ERRα activates its target genes through binding to the ERRE in their promoter regions, and it can bind to its own ERRE, creating a positive transcriptional feedback loop in response to increased energy demands [37]. Luciferase reporter gene assays in cardiomyocytes revealed the ability of Perm1 to directly increase ERRE activity (see Fig 6). Given that the Perm1 protein does not have a consensus sequence for DNA binding but does contain the nuclear export signals and the ØXXLL-type motif that is often involved in protein-protein interaction [10], we speculate that Perm1 acts as a *transcription co-factor* of ERRα. In support of this notion, immunostaining of cardiomyocytes showed that Perm1 is predominantly expressed in the nucleus. In this capacity, Perm1 could presumably increase the positive feedback through ERRα enhancing its own transcription. Given that *Perm1* also contains an ERRE near the transcriptional start site and is activated by ERRα [10], it is plausible that Perm1 can be either upstream or downstream of ERRα. Our study also confirmed the interaction of ERRα with the *Perm1* promoter (S1 Fig). This creates a rather complicated regulatory circuit which potentially confers the metabolic regulatory system with a very high sensitivity to input signals reflecting changing energy demands. Additional studies are needed to determine the molecular mechanism by which Perm1 can directly interact with ERRα, and to elucidate whether ERRα is strictly required for the transcriptional regulation of OXPHOS genes by Perm1.

Our data suggest that Perm1 regultes PGC-1α expression differently at the mRNA and protein levels. Perm1 overexpression decreased the promoter activity of *PGC-1α* (Fig 6C) and the mRNA level of *PGC-1α* (Figs 4A and 5C), suggesting that Perm1 represses the *PGC-1α* gene. On the other hand, PGC-1α protein was increased by Perm1 overexpression (Fig 5D and 5E). This suggests that Perm1 positively regulates PGC-1α expression at the protein level through post-transcriptional modifications (PTM), possibly by stabilizing the PGC-1α protein. We speculate that downregulation of PGC-1α in siPerm1 cardiomyocytes (Fig 3A, 3B and 3G) initially occured at the protein level (Fig 3A and 3B), leading to the reduction of the PGC-1α transcript (Fig 3G) through the autoregulatory feeback loop by which PGC-1α regulates its own expression [38, 39]. It needs to be elucidated whether Perm1 modifies the PGC-1α protein directly or indirectly, by modulating the expression/activity of PTM regulators. Enrichment anlaysis of RNA-seq data showed that Perm1 regulates post-translational protein modification (Fig 3D), which includes protein ubquitination (i.e. Ube2f, Ube2z), sumoylation (i.e. Sumo3), and RAB geranylgeranylation (i.e., Rab23, Rab30, Rab32, Rab40b, Rab6a, Rab9a) (S3 Dataset). Thus, it is plausibile that Perm1 regulates PTM processes by controlling the expression of proteins involved in PTM.

We also noticed that the effect of Perm1 overexpression on the transcription of *PGC-1α* differed between a previous study and our study. AAV-mediated Perm1 overexpression in skeletal muscle led to upregulation of *PGC-1α* [25], while our data shows that Perm1 overexpression in cardiomocytes decreases the mRNA level of *PGC-1α*. It is possible that the repression by Perm1 of the *PGC-1α* gene is cardiac muscle-specific. Previous studies showed that transgenic mice overexpressing PGC-1α in the heart develop dilated cardiomyopathy, in association with the disrupted sarcomeric structure, due to uncontrolled mitochondrial proliferation in the heart [40, 41]. Of note, gain-of-function models with a modest *PGC-1α* overexpression did not induce heart failure [5, 9], suggesting that the development of heart failure in the transgenic mice was due to excessive PGC-1α expression. Hence, in cardiac muscle, PGC-1α must be maintained within an optimal range. It is possible that Perm1 helps to accomplish this task by supressing PGC-1α expression at the transcription level, while stabilizing its protein level through PTMs. Futher studies are necessary to fully understand the role of Perm1 in the complex regulation of PGC-1α expression in the heart [42].

In conclusion, this is the first study demonstrating that Perm1 regulates mitochondrial function in cardiac muscle, possibly by acting as a co-factor of ERRα. Given that Perm1 is a striated muscle-specific protein, similar to Smyd1, while ERRα and PGC-1α are expressed in various tissues, our study suggests that Perm1 provides an additional and muscle-specific regulatory pathway in the ERRα/PGC-1α axis that regulates cardiac energetics.

## Supporting information

S1 Text Supplemental methods, results, discussion, and legend for S1 Fig. (PDF)

S1 Fig DNA Pull-down assay of smyd1 using biotin-labeled perm1 promoter. (PDF)

S1 Table Phenotypic characteristics of C57BL mice subjected to sham treatment, Transverse Aortic Constriction (TAC). (PDF)

S2 Table Clinical data of the study population. (PDF)

S1 Dataset RNA-seq (scr-siRNA vs. siSmyd1 NRVMs). (XLSX)

S2 Dataset RNA-seq (scr-siRNA vs. siPerm1 NRVMs). (XLSX)

S3 Dataset Reactome biological pathways (scr-siRNA vs. siPerm1 NRVMs) (p<0.05). (XLSX)

S4 Dataset KEGG pathway of metabolism regulated by siPerm1. (XLSX)

S5 Dataset Downregulated pathways in siSmyd1 and siPerm1 NRVMs (Reactome). (XLSX)

S6 Dataset Upregulated pathways in siSmyd1 and siPerm1 NRVMs (Reactome). (XLSX)

S1 Raw image (PDF)
