# Supplementary material for: Perm1 regulates cardiac energetics as a downstream target of the histone methyltransferase Smyd1
Source: PLoS One. 2020 Jun 23;15(6):e0234913. doi: 10.1371/journal.pone.0234913 (PMC7310723; doi:10.1371/journal.pone.0234913)
Supplement: S1 Text — (PDF) [file pone.0234913.s001.pdf]

S1 Text

Supplemental Methods

DNA Pull-down Assay

Biotin-labeled Perm1 promoter was prepared by PCR with a biotin-labeled and non-labeled primers as follows: (5’ to 3’): *Perm1 Region 1 (600bp)–* Bitoin-CACATTGTCTGAGATCAAGAGTGATGC and GGCGGTGCCAGGTACTCAAAC; and *Perm1 region 2 (523bp)–* Biotin-GAAGGCTGGTCACCTGCTGG and AGTGACGGCAAGACTTTGGGCC. Primary cultured rat neonatal cardiomyocytes plated on 10 cm dish were lysed with 300 μl of a nuclear lysis buffer [25 mM Hepes, pH7.9, 0.1 mM EDTA, 10 mM Sodium Butyrate, 10 mM NaF, 1 mM DTT, 1 % NP-40 Substitute, 420 mM KCl, 1xProteinase inhibitor cocktail]. The 100 μl lysate was diluted with 300 μl of a dilution buffer [10 mM Hepes, pH 7.9, 2.5 mM MgCl2, 5 % Glycerol, 1 mM DTT, 0.1%NP-40 Substitute]. The total 400 μl of diluted lysate was incubated with 1 μg biotin-labeled DNA and streptavidin-beads at 4 °C for 2-4 hours with rotation. The protein-DNA complex was washed with 1 ml of a washing buffer buffer [10 mM Hepes, pH 7.9, 2.5 mM MgCl2, 5 % Glycerol, and 50 mM KCl 1 mM DTT, 0.1%NP-40 Susbtitute] for 3 times. The protein bound to the DNA was analyzed with Western blot analyses.

Supplemental Results and Discussion

ChIP-PCR showed that Smyd1 interacts with the H3K4me3-rich region of the *Perm1* promoter in the mouse heart (Fig 2E in main text). To examine whether Smyd1 directly binds to this specific region of the *Perm1* promoter, we performed promoter-pulldown assay, using biotin-labeled

double stranded DNA comprising either the H3K4me3-rich promoter region (Region 2) or the region with small H3K4me3 peaks (Region 1) of the *Perm1* promoter (Fig 2D in main text). The transcription factor ERRα and Polymerase II (Pol II) were used as positive controls. As shown in S1 Fig, we observed the binding of Pol II to Region 2, where the transcription start site (TSS) is adjacent, while ERRα bound to both Region 1 and Region 2. In contrast, the binding of Smyd1 to either region could not be detected. Since Smyd1 is a histone modulator and it is currently believed that Smyd1 does not directly bind to DNA [1], the affinity of Smyd1 to the DNA without histone may be limited and the presence of chromatin might be required for Smyd1 to interact with the *Perm1* promoter. Thus, the DNA constructs conjugated with histone might be required to perform *in-vitro* assays. The other possibility is that although Smyd1 binds to the H3K4me3-rich region in the Perm1 promoter, the interaction of Smyd1 requires the other region(s) in Perm1 locus. Thus, in our future study, we will need to conduct reporter gene assays by generating a series of reporter gene constructs that include and exclude H3K4me3-rich region.

Figure Legends

S1 Fig. DNA pull-down assay of Smyd1 using biotin-labeled Perm1 promoter. Biotin-labeled Perm1 promoter was synthesized, targeting Region 1 and Region 2 of the *Perm1* promoter (Top panel). Lysate from neonatal rat ventricular myocytes (NRVMs) were incubated with the biotin-labeled DNA constructs and streptavidin-beads, while the negative control was obtained by incubating with streptavidin-beads but not DNA. Western blotting analysis of the protein bound to the DNA shows that Pol II bound to Region 2 and ERRα to Regions 1 and 2, whereas the binding of Smyd1 to either region was not be detected. PD: pull-down

References

1. Tracy C, Warren JS, Szulik M, Wang L, Garcia J, Makaju A, et al. The Smyd Family of Methyltransferases: Role in Cardiac and Skeletal Muscle Physiology and Pathology. Curr Opin Physiol. 2018;1:140-52. doi: 10.1016/j.cophys.2017.10.001. PubMed PMID: 29435515; PubMed Central PMCID: PMCPMC5807017.
