# Supplementary figures and images for: Perm1 regulates cardiac energetics as a downstream target of the histone methyltransferase Smyd1
Source: PLoS One. 2020 Jun 23;15(6):e0234913. doi: 10.1371/journal.pone.0234913 (PMC7310723; doi:10.1371/journal.pone.0234913)

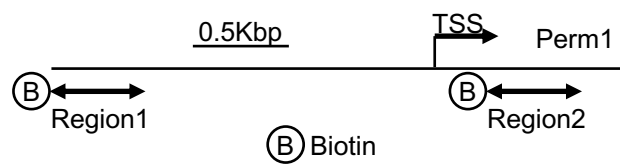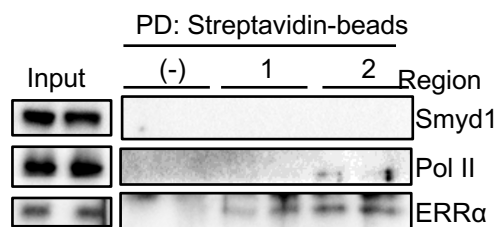

Supplement: S1 Fig — (PDF) [file pone.0234913.s002.pdf]
