# Supplementary material for: Perm1 regulates cardiac energetics as a downstream target of the histone methyltransferase Smyd1
Source: PLoS One. 2020 Jun 23;15(6):e0234913. doi: 10.1371/journal.pone.0234913 (PMC7310723; doi:10.1371/journal.pone.0234913)
Supplement: S1 Table — (PDF) [file pone.0234913.s003.pdf]

S1 Table. Phenotypic Characteristics of C57BL Mice Subjected to Sham Treatment, Transverse Aortic Constriction (TAC)

	Sham (n=4)	TAC (4 weeks) (n=4)
Age, month	3.68 ± 0.49	4.22 ± 0.97
HR, bpm	464 ± 70	443 ± 73
LVEF, %	70.5 ± 6.1	40.3 ± 8.0*
LVFS, %	33.7 ± 4.6	15.9 ± 3.6*
LVEDD, mm	3.07 ± 0.46	3.77 ± 0.50*
LVESD, mm	1.98 ± 0.37	3.12 ± 0.52*
DSEP WT, mm	0.87 ± 0.12	1.40 ± 0.32*
DP WT, mm	0.93 ± 0.15	1.01 ± 0.23
SSEP WT, mm	1.15 ± 0.33	1.57 ± 0.38*
SP WT, mm	1.04 ± 0.20	1.11 ± 0.23

HR, heart rate; LV, left ventricle; EF, ejection fraction, FS, fractional shortening; EDD, end-diastolic dimension; ESD, end-systolic dimension; DSEP, Diastolic septal; DP, Diastolic posterior; SSEP, systolic septal; SP, Systolic posterior; WT, wall thickness. Data are mean ± SD. *P<0.05 vs pre-TAC (paired t test).
