# Supplementary material for: Perm1 regulates cardiac energetics as a downstream target of the histone methyltransferase Smyd1
Source: PLoS One. 2020 Jun 23;15(6):e0234913. doi: 10.1371/journal.pone.0234913 (PMC7310723; doi:10.1371/journal.pone.0234913)
Supplement: S2 Table — (PDF) [file pone.0234913.s004.pdf]

S2 Table. Clinical Data of the Study Population

	Donor (n=7)	***Failing (n=10)***	***p-value***
Age in years, median (range)	42 (31-69)	51 (27-65)	0.86
Gender, male/female	3/4	8/2	
Ischemic cardiomyopathy, n	n/a	6	
Left ventricular ejection fraction, %	64.7 ± 3.1	20 ± 24	<0.05
Cardiac index, L/min/m_2	4.1 ± 0.4	1.9 ± 0.1	<0.05
Mean PAP, mm Hg	No data	34.1 ± 3.3	n/a

PAP: pulmonary artery pressure
