# Supplementary material for: Perm1 regulates cardiac energetics as a downstream target of the histone methyltransferase Smyd1
Source: PLoS One. 2020 Jun 23;15(6):e0234913. doi: 10.1371/journal.pone.0234913 (PMC7310723; doi:10.1371/journal.pone.0234913)
Supplement: S1 Raw image — (PDF) [file pone.0234913.s011.pdf]

Fig 2

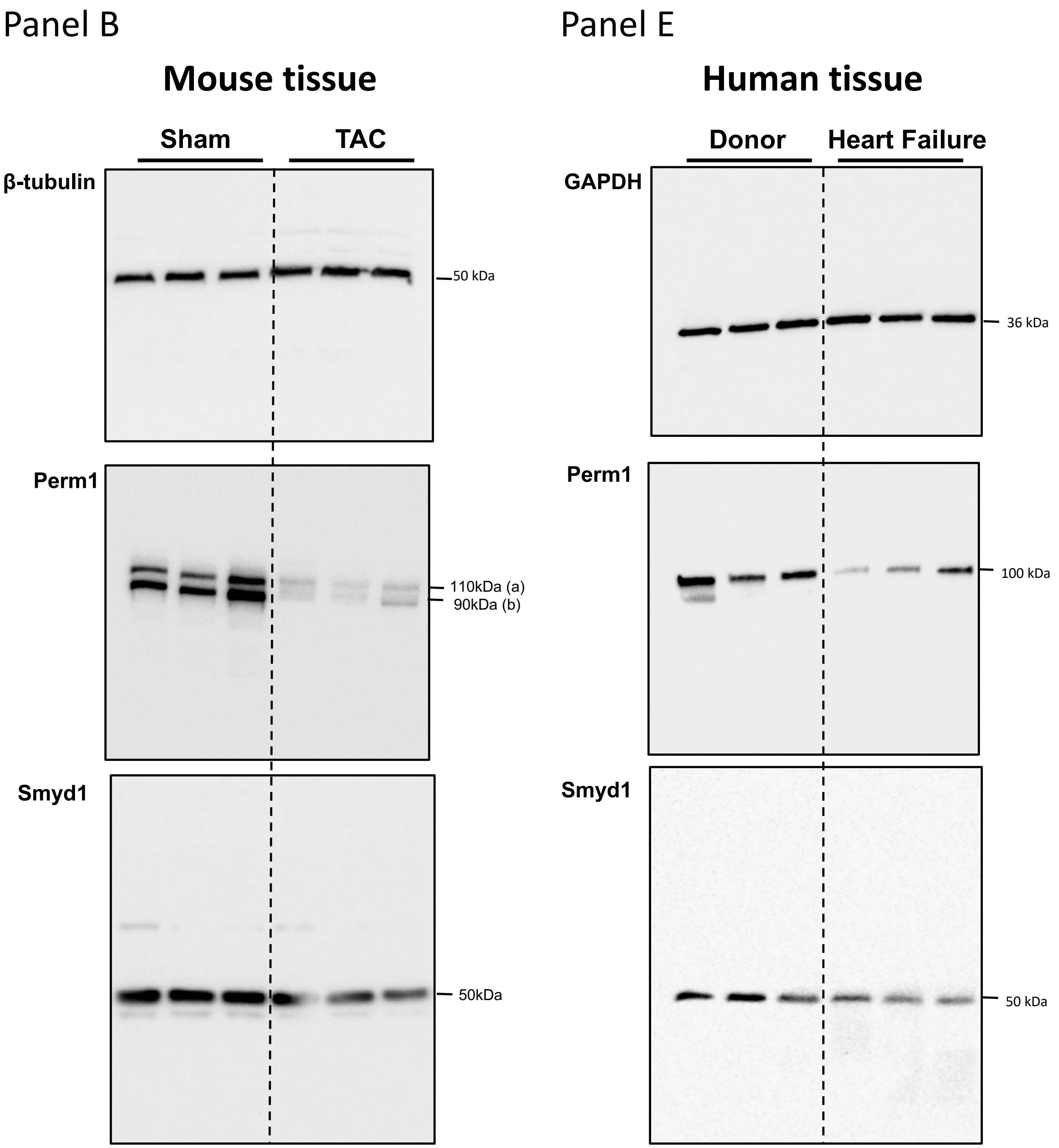

Methods:

The images from western blot analyses were initially obtained using the Image Lab software within the ChemiDoc Bio-Rad Imager. After assuring that no overexposed bands were contained, the collected images were then exported to a TIF file using the Image Lab software. The images in TIF files were inserted into a PowerPoint file to create the figures.

Fig 3A

Samples: Neonatal rat ventricular myocytes (NRVMs)

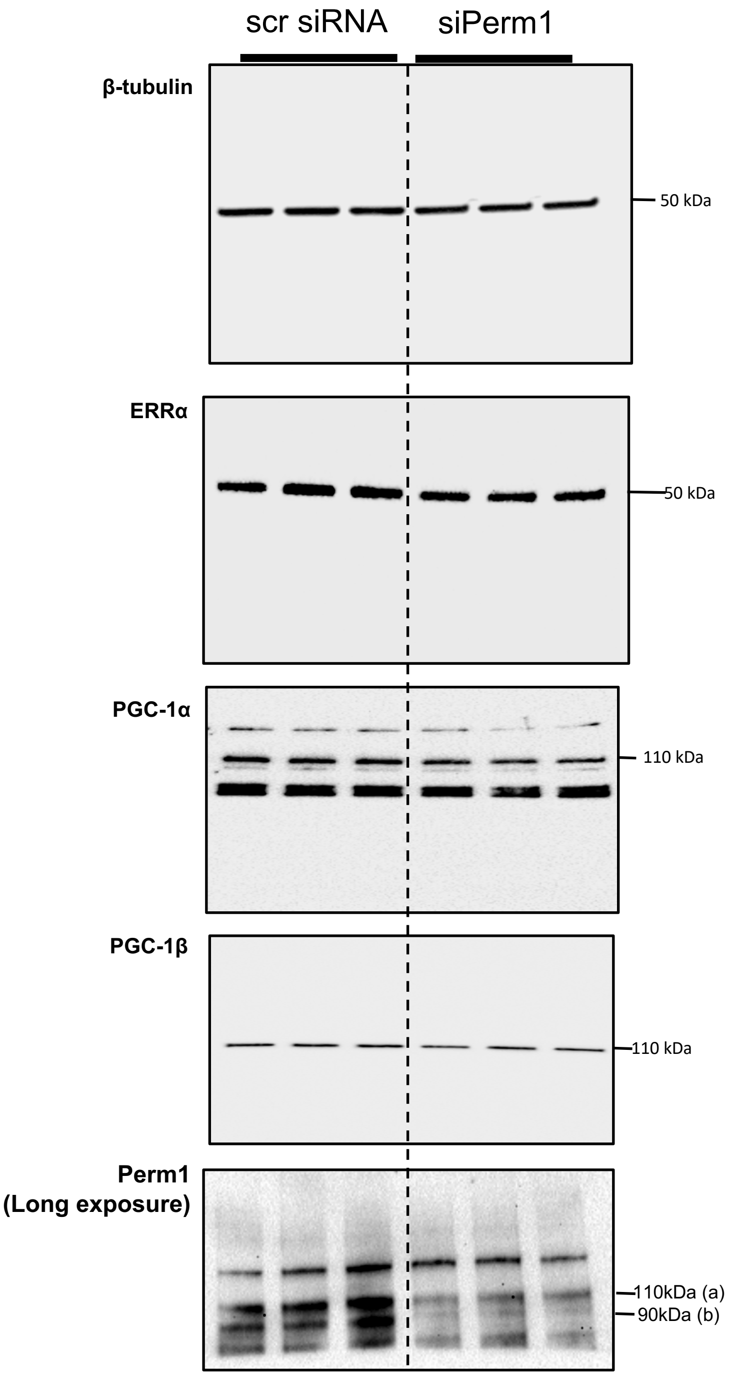

Methods:

The images from western blot analyses were initially obtained using the Image Lab software within the ChemiDoc Bio-Rad Imager. After assuring that no overexposed bands were contained, the collected images were then exported to a TIF file using the Image Lab software. The images in TIF files were inserted into a PowerPoint file to create the figures.

Fig 5D

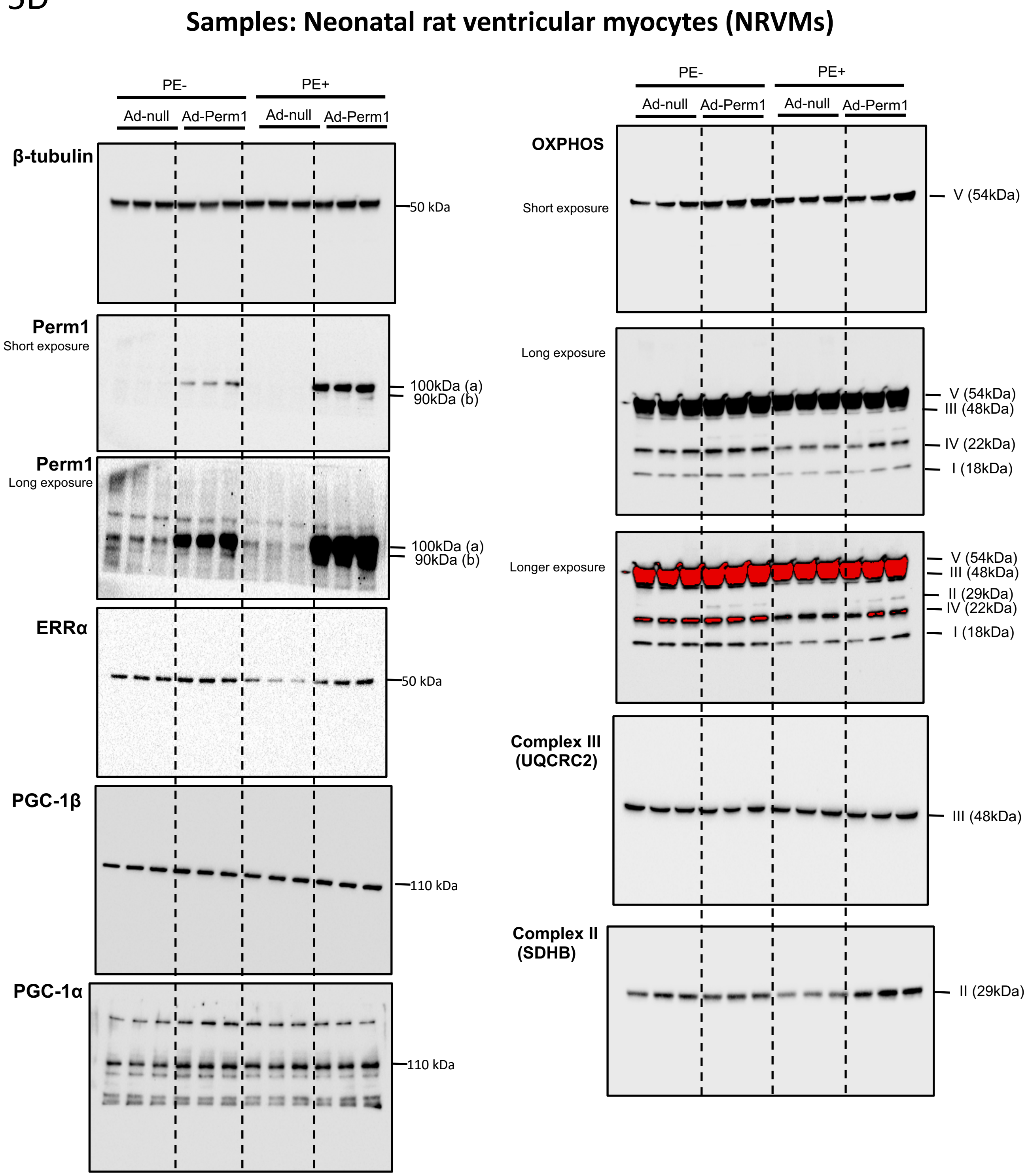

Methods:

The images from western blot analyses were initially obtained using the Image Lab software within the ChemiDoc Bio-Rad Imager. After assuring that no overexposed bands were contained, the collected images were then exported to a TIF file using the Image Lab software. The images in TIF files were inserted into a PowerPoint file to create the figures.
